# Establishment of trimester-specific reference intervals of serum lipids and the associations with pregnancy complications and adverse perinatal outcomes: a population-based prospective study

**DOI:** 10.1080/07853890.2021.1974082

**Published:** 2021-09-09

**Authors:** Yifan Lu, Zhaoxia Jia, Shaofei Su, Lican Han, Lanlan Meng, Guodong Tang, Jing Wang, Chunhong Zhang, Xin Xie, Yi Zhang, Yue Zhang, Yanhong Zhai, Zheng Cao

**Affiliations:** a Department of Laboratory Medicine, Beijing Obstetrics and Gynecology Hospital, Capital Medical University, Beijing, China; b Department of Information and Statistics, Beijing Obstetrics and Gynecology Hospital, Capital Medical University, Beijing, China; c Central Laboratory, Beijing Obstetrics and Gynecology Hospital, Capital Medical University, Beijing, China; d Information Center, Beijing Obstetrics and Gynecology Hospital, Capital Medical University, Beijing, China; e Beijing Maternal and Child Health Care Hospital, Beijing, China

**Keywords:** Lipid, pregnancy, complication, prospective, reference interval

## Abstract

**Background:**

Disturbances in maternal lipid metabolism may increase the risk of developing pregnancy complications and adverse perinatal outcomes. However, there is no consensus as to what constitutes normal serum lipid ranges during pregnancy. Our study was aimed to establish trimester-specific serum lipid reference intervals (RIs) and investigate the associations between maternal dyslipidaemia and adverse outcomes in a population-based study.

**Methods:**

The first- and third-trimester lipid profiles were derived from 16,489 singlet pregnant women for regular antenatal check-ups between 2017 and 2019. The serum samples were assayed for total cholesterol (TC), triglycerides (TG), high-density lipoprotein-cholesterol (HDL-C), and low-density lipoprotein-cholesterol (LDL-C) in the institutional clinical laboratory. The trimester-specific lipid RIs were estimated with both of the direct observational and the indirect Hoffmann methods. The associations between maternal lipid profiling and pregnancy complications and perinatal outcomes were assessed statistically.

**Results:**

Serum levels of TC, TG, LDL-C and HDL-C were all increased significantly in the third trimester of pregnancy. There was no significant difference between the observed RIs established with healthy pregnant women and the calculated RIs derived from the Hoffmann method. A trend towards increased risks of gestational complications and adverse perinatal outcomes was observed in the subjects with elevated levels of TC, TG, and LDL-C or decreased level of HDL-C.

**Conclusions:**

In pregnancy, increased serum levels of TC, TG and LDL-C, and a decreased level of HDL-C posed higher risks of developing pregnancy complications and adverse perinatal outcomes.

**Key messages**
- It is necessary to establish trimester-specific reference intervals for serum lipids including TC, TG, LDL-C and HDL-C that were found significantly increased as the gestational age went up. More importantly, around the upper reference limits of TC, TG and LDL-C (or the lower reference limit of HDL-C), the higher the serum lipid levels were (or the lower the HDL-C level was), the higher risks of developing pregnancy complications and adverse perinatal outcomes were observed.

## Introduction

As one of the most common nutrients required for normal pregnancy, lipid maintains the fundamental needs of developing embryo for key energy and structural components of cells. Hence, an optimum supply of lipids is necessary for proper intrauterine growth of the foetus [1,2]. Complex changes of lipid metabolism were found in pregnant women in whom characteristic features are fat accumulation, increased tissue lipolysis and maternal physiological hyperlipidaemia [2,3].

Elevated serum triglycerides (TG) occur in the early phase of pregnancy as a consequence of increased lipogenesis and suppressed lipolysis [2]. Similar change was also found in total cholesterol (TC), high-density lipoprotein cholesterol (HDL-C) and low-density lipoprotein cholesterol (LDL-C) [4]. As pregnancy progresses into third trimester, the accumulation of fat deposition declines and gradually tails off as a result of both an increased lipolysis and mobilization of triacylglycerols stored in adipose tissue and a decreased activity of adipose tissue lipoprotein lipase [5,6].

Although maternal hyperlipidaemia represents a physiological state during pregnancy, malfunction in the mechanisms regulating lipid metabolic profiles may lead to adverse outcomes [2,7]. Disturbances in maternal lipid metabolism have been shown to increase the risks of pregnancy complications and adverse perinatal outcomes such as preeclampsia (PE), gestational diabetes mellitus (GDM), preterm birth and foetal growth disorders [8,9].

However, there is no consensus as to what constitutes normal serum lipid ranges during pregnancy, which may lead to failure of recognizing potential risks and providing subsequent interventions in a timely fashion. Therefore, it is crucial to establish reference intervals (RIs) for lipids during pregnancy for proper laboratory results interpretation and pathological diagnosis.

The Hoffmann method was first described in 1963 and is an indirect method for RI estimation that does not require enrolment of healthy subjects [10]. Professional groups such as International Federation of Clinical Chemistry Committee on Reference Intervals and Decision Limits highlighted the importance of indirect techniques for RI estimation [11]. In this study, we aimed to establish the trimester-specific RIs of serum lipids, including TG, TC, HDL-C and LDL-C, by recruiting the heathy pregnant women and the indirect Hoffmann method using the existing laboratory data from a relatively large patient cohort. Secondly, we assessed the clinical values of the maternal serum lipids in predicting adverse pregnancy outcomes to fill the gap of risk monitoring of pathological disorders related to lipid metabolism during pregnancy.

## Materials and methods

### Subjects and laboratory data

For the establishment of the trimester-specific RIs of TC, TG, HDL-C and LDL-C, healthy singlet pregnant women aged 20–45 years old who gave a live birth at our institute and presented normal antenatal laboratory workup results (i.e. routine peripheral blood counts, urine and biochemical tests) and normal pre-pregnancy BMI (18.5－23.9) were recruited. All samples were collected at the fasting status. The exclusion criteria were as follows: (i) major organ diseases such as liver, kidney, heart, and lung disease; (ii) positive urine protein; (iii) recent medication, surgery, or other treatments; (iv) acute trauma or acute or chronic inflammation; (v) history of hypertension and diabetes before pregnancy; (vi) other complications, for example, ectopic pregnancy, gestational diabetes, or gestational hypertension (including preeclampsia, eclampsia, chronic hypertension with pregnancy, chronic hypertension with preeclampsia, or haemolysis, elevated liver enzymes, low platelet count syndrome); (vii) infectious diseases such as hepatitis B virus, hepatitis C virus and human immunodeficiency virus.

For the association study, the serum lipid testing results were collected from the outpatients who visited the Beijing Obstetrics and Gynaecology Hospital for regular antenatal check-ups. From October of 2017 to October of 2019, totally 16,489 participants that were singlets with complete lipid testing records and had given a live birth were enrolled in the subsequent statistical analyses. All the participants’ medical records were retrospectively reviewed for the subsequent association studies. Considering the completeness of the medical records and the relevance with serum lipids, four pregnant complications including gestational hypertension (GH), gestational diabetes mellitus (GDM), preeclampsia (PE) and intrahepatic cholestasis of pregnancy (ICP), and two adverse pregnant outcomes including foetal macrosomia and postpartum haemorrhage (PPH) were analysed and used as grouping conditions in the following study. The study protocol was approved by the Ethics Committee of Beijing Obstetrics and Gynaecology Hospital (2017-KY-078-01). The need for informed consent from included individuals was waived by the Ethics Committee as all the lipid tests were part of usual patients’ care in their pregnancy and the laboratory data was anonymized before its use.

### Experiment methods

The maternal levels of TC, TG, HDL-C and LDL-C were assayed on the fully automated ARCHITECT ci16200 Integrated System Chemistry/Immunology Analyser (Abbott Park, IL, USA) using cholesterol assay kit (H05119R02, Abbott Park, IL, USA), triglyceride assay kit (G07893R02, Abbott Park, IL, USA), LDL assay kit (G69452R13, Abbott Park, IL, USA) and HDL assay kit (G05251R03, Abbott Park, IL, USA). The collected serum samples of the recruited subjects were stored at 4 °C for less than 24 h or at −20 °C for longer time before testing.

The limit of detection for serum TC, TG, HDL-C and LDL-C were 0.03 mmol/L, 0.02 mmol/L, 0.06 mmol/L and 0.03 mmol/L, respectively. The intra-assay coefficients of variation (CV) of serum TC, TG, HDL-C and LDL-C were 0.6% to 0.8%, 0.7% to 0.8%, 1.0% to 1.7%, and 1.1% to 1.4%, respectively; the inter-assay CV were 0.4% to 0.8%, 0.4% to 0.6%, 0.5% to 1.1%, and 2.2%, respectively.

### Statistical analysis

The Kolmogorov–Smirnov test was used to evaluate the normality of the data distribution. Numerical values were expressed as the mean and standard deviation (SD) for variables with normal distribution and as the median and percentiles for nonnormally distributed data. To examine the statistical significance of serum lipid levels between first and third trimesters, the Mann-Whitney U-test was used and *p* < .05 was considered to be statistically significant. The RIs for the four lipids, derived from the healthy pregnant women (the same patients used for first and third trimesters RIs), were estimated by the IBM SPSS Statistic 21 (SPSS Inc., Chicago, IL, USA, RRID:SCR_002865) using the nonparametric approach. At the same time, the Hoffmann method [10,12] was used to calculate the RIs with the trimester-specific lipid profiling results using the relevant laboratory data of the 16,489 pregnant women. The detailed steps for the estimation of RIs with the Hoffmann method can be found in the protocol published previously [12,13]. Briefly, the data were entered in an MS Excel spreadsheet (Microsoft Corporation, WA, USA). The results of the lipid tests for a specific trimester were logarithmically transformed and then sorted in an ascending order. Further, the formula = NORM.INV was applied to generate a new set of distribution numbers to create a quantile–quantile plot (Q–Q plot) with the log-transformed data. The linear portion of the Q–Q plot was visually examined, and the Y values were calculated when X was equal to −2 and 2, using the best fit linear regression equation. With a standard normal distribution, the interval between μ ± 2σ represents the central 95% (2.5–97.5%) range of the measurements or a particular RI (μ represents the mean and σ represents the standard deviation). To determine the statistical significance of the differences between directly observed RIs and the indirectly estimated RIs, the reference change value (RCV) was calculated as previously described [14].

Additionally, the multivariate logistic regression analysis, odds ratios (ORs) and 95% confidence intervals (CIs) were calculated, while maternal age and pre-pregnancy body mass index (BMI) were adjusted as confounders. Basically, the two variables of age and pre-pregnancy BMI were included as covariates into the logistic regression analysis, to calculate the resulting OR and *p* values for the adjusted risk relationship between serum lipids and outcomes. In the logistic analysis, as the measurements of serum lipids were converted into binary variables according to the established RIs, log transformation for the lipid data was not performed.

The receiver operating characteristics (ROC) curve was used to analyse the predictive values of the four lipids for adverse pregnancy outcomes. Sensitivity, specificity and cut-off values were reported when Youden’s index was at the maximum, which was used to calculate positive predictive value (PPV) and negative predictive value (NPV). The above statistical calculations were carried out with the SPSS software.

## Results

**Figure 1.**
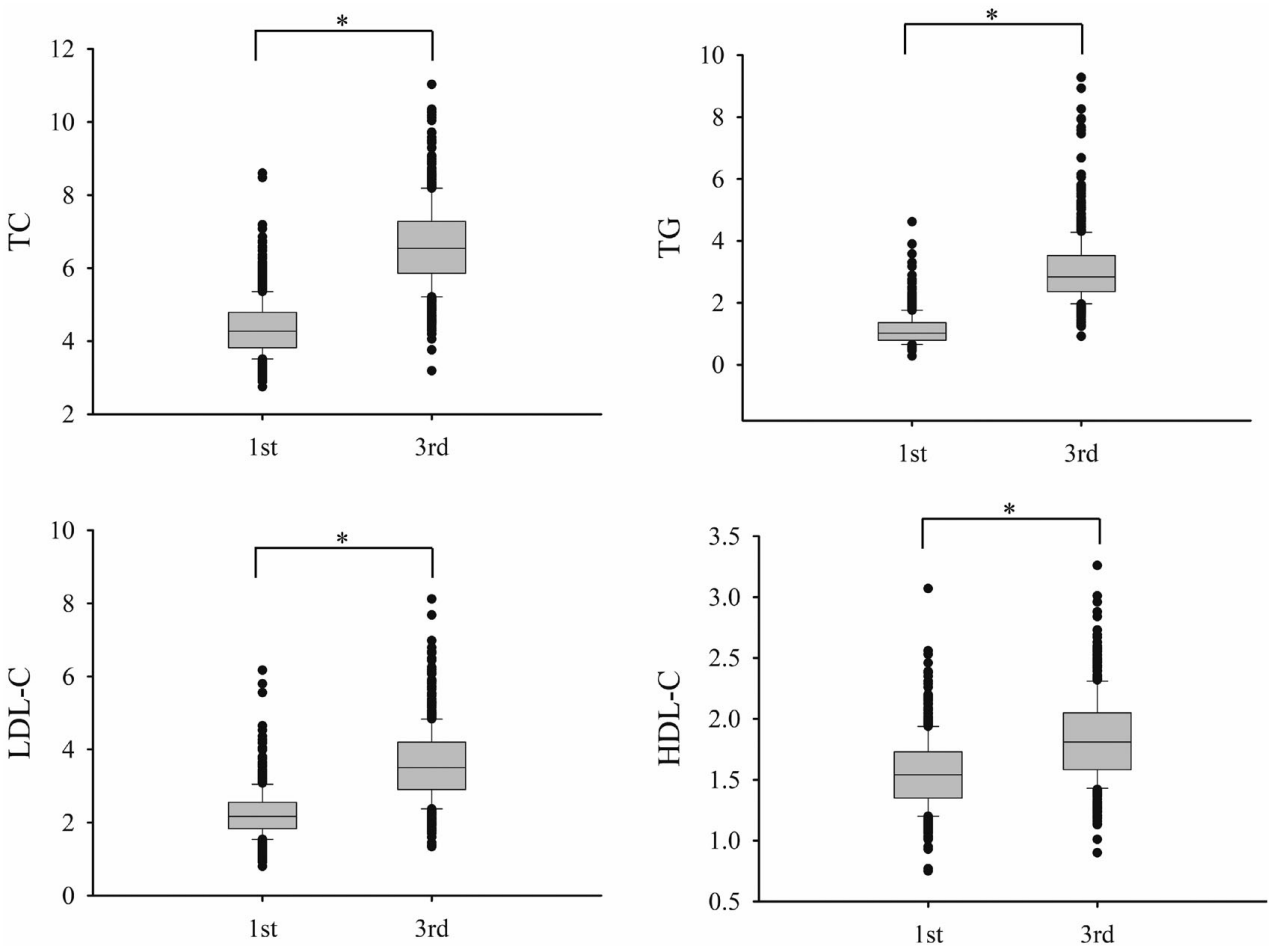
The box plots of serum lipid levels in healthy pregnant women in first and third trimesters. *Indicates *p* < .001. TC: total cholesterol; TG: triglycerides; HDL-C: high-density lipid cholesterol; LDL-C: low-density lipid cholesterol.

According to the CLSI guideline C28-A3 [15], totally 603 healthy pregnant women meeting the recruiting criteria, with the laboratory data of both of the first and third trimester serum lipids from the same subjects, were enrolled for trimester-specific RIs establishment. Figure 1 showed the overall data distribution for TC, TG, HDL-C and LDL-C. The serum TC, TG, HDL-C and LDL-C were all significantly elevated in the third trimester than those in the first trimester (*p* < .001). The trimester-specific RIs for serum lipids tests determined with non-parametric analysis were shown in Table 1. At the same time, the indirect RI estimation method (Hoffmann method), which was developed for relatively large data set and allowed mixture with “unhealthy subjects”, was applied with the outpatients’ (*n* = 16,489) lipid tests data. As shown in Table 1, no significant difference was found between the observed RIs with healthy pregnant women and the calculated RIs derived from the Hoffmann method (absolute difference % smaller than RCV %).

**Table 1.** Reference intervals of serum lipids in the first and third trimesters.

	First trimester			Third trimester			RCV, %
	95% reference interval, mmol/L	Hoffmann method, mmol/L	Absolute difference, %	95% reference interval, mmol/L	Hoffmann method, mmol/L	Absolute difference, %	
TC	3.11–6.11	3.07–5.82	1.30–4.98	4.56–9.31	4.50–9.02	1.33–3.22	10.31
TG	0.54–2.33	0.48–2.20	12.50–5.91	1.68–5.67	1.53–5.52	9.80–2.72	20.79
HDL-C	1.06–2.18	1.00–2.18	6.00–0	1.23–2.56	1.19–2.64	3.36–3.03	13.91
LDL-C	1.26–3.80	1.30–3.61	3.08–5.26	1.93–5.90	1.97–5.84	2.03–1.03	13.67

TC: total cholesterol; TG: triglycerides; HDL-C: high-density lipid cholesterol; LDL-C: low-density lipid cholesterol.

Additionally, the predictive values of serum lipids were examined with the cohort of 16,489 pregnant women with relevant antenatal laboratory workup data and associated medical records through delivery. Due to the nature and development timing of these diseases, the prediction of GH, GDM, PE and ICP was only assessed with the first trimester serum lipids; the prediction of macrosomia and PPH was conducted with the first and third trimester serum lipids separately. The clinical diagnosis criteria for the above complications and adverse outcomes were listed in Supplementary Table 1.

The demographic data of age, pre-pregnancy BMI and the basic statistics of serum lipids were summarized in Supplementary Table 2. Clearly, the first trimester TC, TG and LDL-C were significantly increased, while the HDL-C were significantly decreased in GH, GDM, PE and PPH group *(p* < .001). Similar changes, for TG and HDL-C, were also found in the macrosomia group. In the assessment of third trimester serum lipids, the decreasing tendency of HDL-C was observed in both of the macrosomia and PPH groups. The third trimester TG level in the subjects developing macrosomia and PPH were significantly increased (Supplementary Table 2).

**Figure 2.**
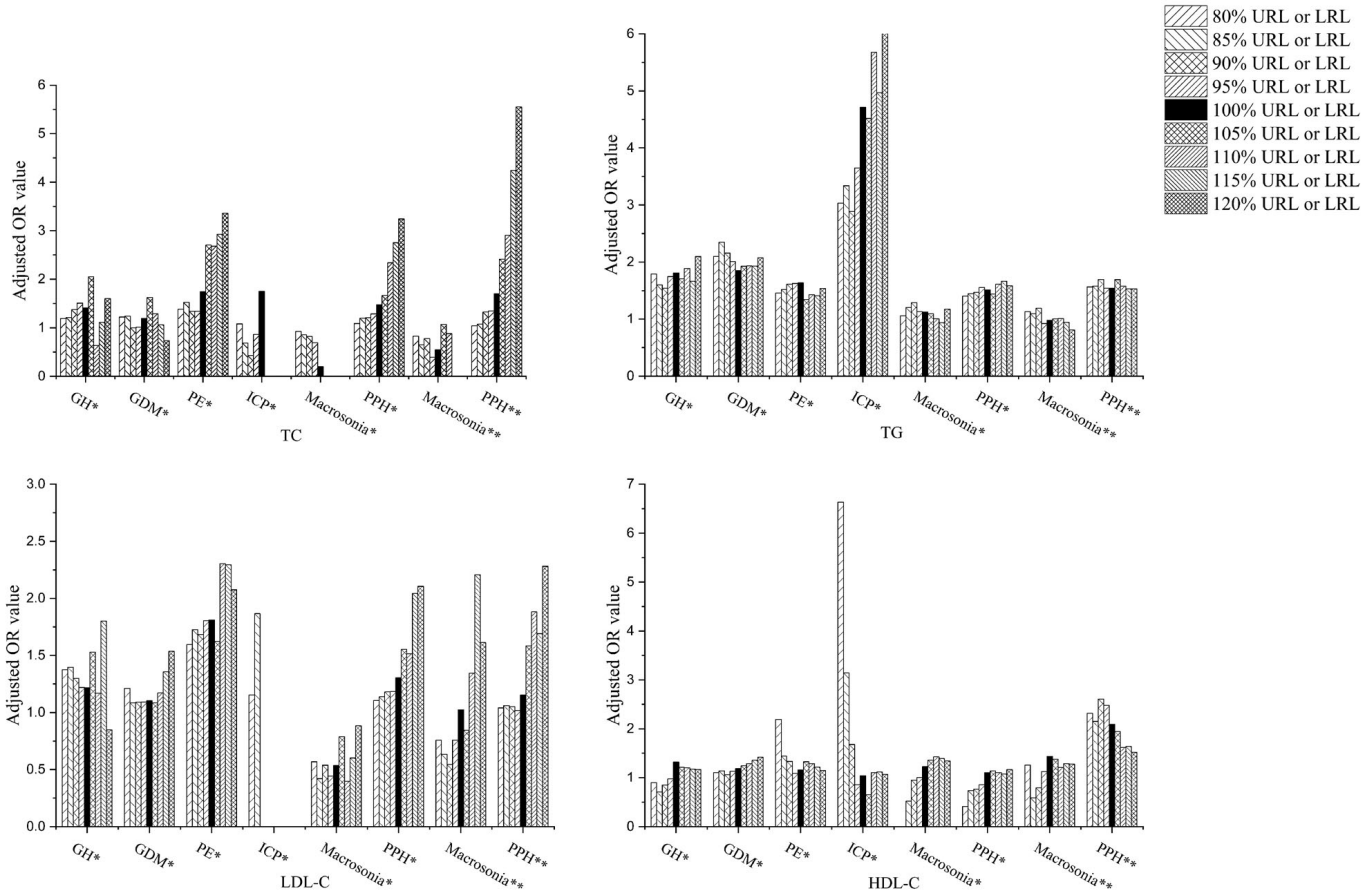
The column diagrams of adjusted OR value determined at different lipid serum levels. *: first trimester; **: third trimester; GH: gestational hypertension; GDM: gestational diabetes mellitus; PE: preeclampsia; ICP: intrahepatic cholestasis of pregnancy; PPH: postpartum haemorrhage; TC: total cholesterol; TG: triglycerides; HDL-C: high-density lipid cholesterol; LDL-C: low-density lipid cholesterol; URL: upper reference interval limit, used in the OR analysis for TC, TG and LDL-C; LRL: lower reference interval limit, used in the OR analysis for HDL-C.

In the ROC analyses, the AUCs of all the serum lipids ranged between 0.50 and 0.66 (Table 2 and Supplementary Figure 1). With the cut-off values obtained with the Youden Index in the ROC curves, the PPVs were between 0.3% and 12.0%, and the NPVs were between 92.7% and 99.9% (Table 2), demonstrating a far better performance in ruling out than ruling in those disease conditions. To further investigate the impact of gestational lipid levels on pregnancy complications or perinatal outcomes, the logistical regression analysis was performed with age and pre-pregnancy BMI as covariates (Tables 3, 4 and Figure 2). For the purpose of risk assessment, the OR values were calculated with the cut-offs set around the upper reference limits (URLs) for TC, TG, and LDL-C. By contrast, the lower reference limits (LRLs) were used for the OR calculation for HDL-C.

**Table 2.** ROC analysis of predictive value of serum lipids for pregnancy complications and adverse pregnancy outcomes.

	AUC (95% CI)	Cut-off value	PPV (%)	NPV (%)
GH				
TC*	0.56 (0.55–0.57)	4.07	2.3	98.6
TG*	0.62 (0.61–0.63)	1.39	3.8	98.6
HDL-C*	0.58 (0.57–0.58)	1.32	2.7	98.4
LDL-C*	0.58 (0.58–0.59)	2.10	2.4	98.7
GDM				
TC*	0.57 (0.55–0.58)	4.59	9.9	93.5
TG*	0.66 (0.64–0.67)	1.12	12.0	95.4
HDL-C*	0.58 (0.56–0.59)	1.40	9.7	93.9
LDL-C*	0.58 (0.56–0.60)	2.29	9.7	94.0
PE				
TC*	0.59 (0.57–0.61)	4.19	4.8	97.3
TG*	0.63 (0.61–0.65)	1.14	6.0	97.4
HDL-C*	0.58 (0.56–0.60)	1.39	5.2	97.0
LDL-C*	0.62 (0.60–0.65)	2.25	5.5	97.5
ICP				
TC*	0.54 (0.45–0.63)	4.13	0.3	99.8
TG*	0.53 (0.44–0.61)	1.89	0.7	99.8
HDL-C*	0.59 (0.52–0.67)	1.54	0.3	99.9
LDL-C*	0.52 (0.44–0.61)	2.19	0.3	99.8
Macrosomia				
TC*	0.5 (0.47–0.53)	4.71	2.1	98.3
TG*	0.56 (0.53–0.59)	0.97	2.1	98.6
HDL-C*	0.57 (0.53–0.60)	1.48	2.2	98.7
LDL-C*	0.52 (0.49–0.55)	2.18	2.0	98.5
TC**	0.55 (0.52–0.58)	6.56	2.0	98.6
TG**	0.57 (0.54–0.60)	2.46	2.1	98.9
HDL-C**	0.58 (0.55–0.61)	1.73	2.3	98.6
LDL-C**	0.55 (0.52–0.58)	3.33	2.1	98.5
PPH				
TC*	0.53 (0.52–0.55)	4.43	8.7	93.1
TG*	0.56 (0.55–0.58)	1.01	9.0	93.8
HDL-C*	0.54 (0.52–0.56)	1.51	8.5	93.6
LDL-C*	0.54 (0.53–0.56)	2.37	9.0	93.2
TC**	0.52 (0.50–0.54)	6.17	8.3	92.9
TG**	0.57 (0.55–0.58)	3.04	9.4	93.8
HDL-C**	0.58 (0.56–0.60)	1.70	9.7	93.9
LDL-C**	0.52 (0.50–0.54)	2.57	9.1	92.7

*: first trimester; **: third trimester; BMI: body mass index; CI: confidence interval; GH: gestational hypertension; GDM: gestational diabetes mellitus; HDL-C: high-density lipid cholesterol; ICP: intrahepatic cholestasis of pregnancy; LDL-C: low-density lipid cholesterol; PE: preeclampsia; PPH: postpartum haemorrhage; TC: total cholesterol; TG: triglycerides.

**Table 3.** Logistic regression analysis of the risk of serum TC and TG for pregnancy complications and adverse perinatal outcomes.

	GH*		GDM*		PE*		ICP*	
	OR (95% CI)	*p* value	OR (95% CI)	*p* value	OR (95% CI)	*p* value	OR (95% CI)	*p* value
TC								
80% URL	1.19 (0.91–1.56)	.21	1.22 (1.06–1.41)	<.01	1.38 (1.14–1.66)	<.01	1.08 (0.50–2.36)	.85
85% URL	1.21 (0.87–1.69)	.26	1.24 (1.04–1.47)	.02	1.52 (1.22–1.90)	<.01	0.69 (0.21–2.24)	.53
90% URL	1.37 (0.91–2.07)	.13	1.00 (0.79–1.27)	.98	1.34 (1.00–1.80)	.05	0.43 (0.06–3.12)	.40
95% URL	1.51 (0.89–2.57)	.13	1.01 (0.73–1.40)	.94	1.34 (0.90–1.99)	.15	0.86 (0.12–6.30)	.88
100% URL	1.41 (0.66–3.04)	.38	1.19 (0.78–1.84)	.42	1.75 (1.06–2.89)	.03	1.75 (0.24–12.83)	.58
105% URL	2.05 (0.82–5.10)	.12	1.62 (0.94–2.81)	.08	2.70 (1.48–4.95)	<.01	0	.99
110% URL	0.63 (0.09–4.60)	.65	1.29 (0.60–2.74)	.51	2.68 (1.24–5.80)	.01	0	.99
115% URL	1.11 (0.15–8.19)	0.92	1.06 (0.37–3.05)	0.91	2.93 (1.11–7.74)	0.03	0	.99
120% URL	1.60 (0.21–12.03)	.65	0.74 (0.17–3.20)	.68	3.36 (1.10–10.25)	.03	0	.99
TG								
80% URL	1.79 (1.31–2.45)	<.01	2.10 (1.78–2.47)	<.01	1.46 (1.16–1.84)	<.01	3.04 (1.34–6.89)	<.01
85% URL	1.60 (1.13–2.27)	<.01	2.34 (1.97–2.79)	<.01	1.52 (1.18–1.95)	<.01	3.34 (1.41–7.90)	<.01
90% URL	1.54 (1.04–2.28)	.03	2.16 (1.78–2.62)	<.01	1.61 (1.23–2.11)	<.01	2.89 (1.08–7.70)	.03
95% URL	1.74 (1.16–2.63)	<.01	2.01 (1.62–2.49)	<.01	1.62 (1.21–2.17)	<.01	3.65 (1.36–9.76)	.01
100% URL	1.81 (1.16–2.82)	<.01	1.85 (1.46–2.35)	<.01	1.63 (1.19–2.25)	<.01	4.71 (1.76–12.64)	<.01
105% URL	1.71 (1.04–2.81)	.04	1.93 (1.48–2.50)	<.01	1.34 (0.92–1.95)	.12	4.52 (1.53–13.31)	.01
110% URL	1.89 (1.12–3.20)	.02	1.93 (1.45–2.57)	<.01	1.43 (0.95–2.14)	.08	5.68 (1.92–16.76)	<.01
115% URL	1.67 (0.91–3.04)	.10	1.93 (1.41–2.64)	<.01	1.41 (0.91–2.19)	.13	4.97 (1.46–16.88)	.01
120% URL	2.10 (1.15–3.84)	.02	2.08 (1.48–2.92)	<.01	1.54 (0.96–2.46)	.07	6.16 (1.81–20.96)	<.01
TC								
80% URL	0.92 (0.69–1.25)	.60	1.09 (0.94–1.26)	.26	0.83 (0.59–1.15)	.26	1.04 (0.89–1.21)	.60
85% URL	0.86 (0.58–1.26)	.43	1.20 (1.00–1.43)	.05	0.65 (0.40–1.05)	.08	1.07 (0.89–1.30)	.46
90% URL	0.82 (0.49–1.37)	.46	1.21 (0.96–1.52)	.11	0.78 (0.42–1.43)	.41	1.32 (1.04–1.68)	.02
95% URL	0.69 (0.32–1.48)	.34	1.29 (0.94–1.75)	.11	0.39 (0.13–1.24)	.11	1.34 (0.98–1.85)	.07
100% URL	0.20 (0.03–1.42)	.11	1.48 (0.98–2.22)	.06	0.54 (0.13–2.20)	.39	1.70 (1.13–2.56)	.01
105% URL	0	.98	1.66 (0.96–2.88)	.07	1.07 (0.26–4.36)	.93	2.42 (1.47–3.96)	<.01
110% URL	0	.97	2.34 (1.24–4.40)	.01	0.88 (0.12–6.36)	.90	2.90 (1.58–5.32)	<.01
115% URL	0	.97	2.75 (1.26–6.04)	.01	0	.97	4.24 (2.06–8.72)	<.01
120% URL	0	.97	3.24 (1.28–8.21)	.01	0	.98	5.55 (1.93–15.94)	<.01
TG								
80% URL	1.06 (0.73–1.53)	.77	1.40 (1.17–1.69)	<.01	1.13 (0.79–1.63)	.51	1.56 (1.32–1.85)	<.01
85% URL	1.20 (0.81–1.78)	.35	1.45 (1.18–1.77)	<.01	1.10 (0.72–1.66)	.67	1.58 (1.31–1.91)	<.01
90% URL	1.29 (0.84–1.96)	.24	1.47 (1.18–1.83)	<.01	1.19 (0.76–1.87)	.45	1.70 (1.38–2.09)	<.01
95% URL	1.13 (0.70–1.83)	.62	1.56 (1.22–1.98)	<.01	0.92 (0.53–1.62)	.78	1.54 (1.21–1.96)	<.01
100% URL	1.12 (0.66–1.92)	.67	1.51 (1.16–1.98)	<.01	0.98 (0.53–1.80)	.94	1.55 (1.18–2.02)	<.01
105% URL	1.09 (0.60–1.98)	.77	1.44 (1.07–1.94)	.01	1.00 (0.51–1.97)	.99	1.69 (1.27–2.25)	<.01
110% URL	1.01 (0.51–1.99)	.99	1.61 (1.17–2.22)	<.01	1.01 (0.47–2.16)	.98	1.58 (1.13–2.20)	.01
115% URL	0.94 (0.43–2.02)	.87	1.67 (1.18–2.35)	<.01	0.94 (0.52–1.69)	.84	1.53 (1.19–1.97)	<.01
120% URL	1.17 (0.54–2.53)	.69	1.58 (1.08–2.33)	.02	0.81 (0.30–2.20)	.68	1.53 (1.03–2.28)	.03

*: first trimester; **: third trimester; CI: confidence interval; GH: gestational hypertension; GDM: gestational diabetes mellitus; ICP: intrahepatic cholestasis of pregnancy; LRL: lower reference interval limit; OR: odds ratio; PE: preeclampsia; PPH: postpartum haemorrhage; TC: total cholesterol; TG: triglycerides; URL: upper reference interval limit.

As shown in Tables 3 and 4, increased risk of developing GH was seen if serum TG level was higher than its URL (OR: 1.81, 95% CI: 1.16–2.82). However, the ORs were not essentially changed when the cut-offs were ranged between 80% to 120% of URLs for TG. Similar observation was made in GDM, the risk of which was increased when the serum TG level was higher than its URL (OR: 1.85, 95% CI: 1.46–2.35) but not further elevated with the cut-offs changed between 80%–120% URL. Further, it was shown that the first-trimester TC (OR:1.75, 95% CI: 1.06–2.89), TG (OR: 1.63, 95% CI: 1.19–2.25) or LDL-C (OR: 1.81, 95% CI: 1.14–2.88) levels higher than their URLs significantly increased the risk of PE development. More importantly, the OR value was almost tripled from 1.38 (95% CI: 1.14–1.66) to 3.36 (95% CI: 1.10–10.25) when the TC cut-off value was incremented from 80% to 120% of its URL. Likewise, if the serum TG level was increased from 80% to 120% of its URL, the risk for pregnant women developing ICP was ramped up more than twice as much, with OR values changed from 3.04 (95% CI: 1.34–6.89) to 6.16 (95% CI: 1.81–20.96). Similar to what was observed with PE, the risk of presenting PPH after delivery was sensitive and gradually increased along with the incrementation of serum TC levels of both first and third trimesters. For instance, the OR values was elevated from 1.70 (95% CI: 1.13–2.56) to 5.55 (95% CI: 1.93–15.94) when the cut-off set for third-trimester serum TC was increased from 100% to 120% URL. In addition, the patients with serum TG higher than its URL or HDL-C lower than the LRL also displayed moderately increased risk for PHP. Neither first- nor third-trimester serum lipids were associated with the macrosomia during pregnancy. Interestingly, both older age and increased pre-pregnancy BMI were associated with elevated risks of developing GDM, PE, macrosomia and PPH (Supplementary Table 3). Increased risk for GH in pregnancy was associated with pre-pregnancy BMI, but not age (Supplementary Table 3).

**Table 4.** Logistic regression analysis of the risk of serum HDL-C and LDL-C for pregnancy complications and adverse perinatal outcomes.

	GH*		GDM*		PE*		ICP*	
LDL-C	OR (95% CI)	*p* value	OR (95% CI)	*p* value	OR (95% CI)	*p* value	OR (95% CI)	*p* value
80% URL	1.37 (0.99–1.91)	.06	1.21 (1.01–1.46)	.04	1.60 (1.28–2.00)	<.01	1.15 (0.41–3.29)	.79
85% URL	1.40 (0.95–2.05)	.09	1.08 (0.87–1.36)	.49	1.73 (1.34–2.23)	<.01	1.87 (0.65–5.34)	.24
90% URL	1.30 (0.80–2.10)	.28	1.09 (0.83–1.43)	.53	1.68 (1.24–2.29)	<.01	0	.98
95% URL	1.22 (0.66–2.26)	.53	1.09 (0.77–1.54)	.61	1.80 (1.24–2.63)	<.01	0	.99
100% URL	1.22 (0.56–2.62)	.62	1.10 (0.72–1.70)	.66	1.81 (1.14–2.88)	.01	0	.99
105% URL	1.53 (0.66–3.52)	.32	1.08 (0.64–1.83)	.76	1.62 (0.91–2.89)	.10	0	.99
110% URL	1.17 (0.37–3.73)	.79	1.17 (0.63–2.18)	.62	2.31 (1.22–4.34)	.01	0	.99
115% URL	1.80 (0.56–5.83)	.33	1.36 (0.65–2.82)	.41	2.30 (1.06–4.99)	.04	0	.99
120% URL	0.85 (0.12–6.22)	.87	1.54 (0.67–3.54)	.31	2.08 (0.79–5.45)	.14	0	.99
HDL-C								
80% LRL	0.90 (0.22–3.75)	.89	1.11 (0.54–2.29)	.78	2.19 (1.08–4.46)	.03	6.63 (0.87–50.40)	.07
85% LRL	0.71 (0.22–2.26)	.56	1.14 (0.67–1.92)	.63	1.44 (0.79–2.61)	.23	3.14 (0.42–23.52)	.27
90% LRL	0.85 (0.37–1.94)	.70	1.06 (0.70–1.61)	.77	1.33 (0.83–2.15)	.24	1.68 (0.23–12.45)	.61
95% LRL	0.98 (0.55–1.73)	.94	1.13 (0.84–1.52)	.42	1.09 (0.75–1.60)	.65	0.85 (0.12–6.30)	.88
100% LRL	1.32 (0.88–1.99)	.18	1.19 (0.94–1.50)	.16	1.16 (0.86–1.57)	.34	1.04 (0.25–4.40)	.96
105% LRL	1.21 (0.85–1.73)	.29	1.25 (1.03–1.51)	.03	1.32 (1.04–1.69)	.03	0.65 (0.16–2.75)	.56
110% LRL	1.20 (0.88–1.63)	.24	1.29 (1.09–1.52)	<.01	1.29 (1.04–1.59)	.02	1.11 (0.43–2.88)	.84
115% LRL	1.17 (0.89–1.55)	.25	1.35 (1.17–1.57)	<.01	1.22 (1.00–1.48)	.05	1.12 (0.49–2.57)	.79
120% LRL	1.17 (0.91–1.51)	.23	1.42 (1.24–1.62)	<.01	1.15 (0.96–1.36)	.14	1.07 (0.50–2.28)	.86
LDL-C								
80% URL	0.57 (0.36–0.90)	.02	1.11 (0.91–1.34)	.31	0.76 (0.48–1.19)	.22	1.04 (0.86–1.26)	.68
85% URL	0.42 (0.22–0.80)	.01	1.14 (0.90–1.43)	.27	0.63 (0.34–1.16)	.14	1.06 (0.84–1.34)	.62
90% URL	0.54 (0.26–1.10)	.09	1.18 (0.89–1.56)	.24	0.54 (0.24–1.23)	.14	1.05 (0.78–1.41)	.74
95% URL	0.44 (0.16–1.20)	.11	1.18 (0.83–1.68)	.34	0.76 (0.31–1.85)	.54	1.02 (0.70–1.48)	.93
100% URL	0.54 (0.17–1.69)	.29	1.30 (0.85–2.00)	.22	1.02 (0.38–2.78)	.96	1.15 (0.73–1.83)	.55
105% URL	0.79 (0.23–2.50)	.69	1.55 (0.96–2.51)	.07	0.84 (0.21–3.44)	.81	1.58 (0.94–2.67)	.09
110% URL	0.40 (0.05–2.87)	.36	1.51 (0.84–2.73)	.17	1.34 (0.33–5.52)	.68	1.88 (1.02–3.46)	.04
115% URL	0.60 (0.08–4.39)	.62	2.04 (1.06–3.94)	.03	2.21 (0.53–9.12）	.27	1.69 (0.77–3.72)	.19
120% URL	0.88 (0.12–6.49)	.90	2.11 (0.97–4.55)	.06	1.61 (0.22–11.84)	.64	2.28 (0.96–5.45)	.06
HDL-C								
80% LRL	0	.97	0.41 (0.13–1.33)	.14	1.26 (0.30–5.18)	.75	2.32 (1.26–4.23)	.01
85% LRL	0.52 (0.13–2.13)	.36	0.74 (0.38–1.41)	.36	0.59 (0.14–2.40)	.46	2.15 (1.39–3.33)	<.01
90% LRL	0.95 (0.42–2.17)	.90	0.77 (0.47–1.25)	.28	0.79 (0.29–2.15)	.65	2.61 (1.86–3.65)	<.01
95% LRL	1.00 (0.55–1.81)	1.00	0.85 (0.61–1.20)	.36	1.12 (0.57–2.21)	.74	2.48 (1.89–3.26)	<.01
100% LRL	1.23 (0.79–1.90)	.36	1.11 (0.86–1.42)	.43	1.44 (0.88–2.34)	.14	2.09 (1.66–2.64)	<.01
105% LRL	1.36 (0.95–1.95)	.09	1.14 (0.93–1.39)	.22	1.38 (0.92–2.07)	.12	1.94 (1.60–2.36)	<.01
110% LRL	1.42 (1.05–1.93)	.02	1.11 (0.93–1.31)	.25	1.21 (0.84–1.73)	.31	1.62 (1.36–1.92)	<.01
115% LRL	1.39 (1.06–1.83)	.02	1.08 (0.93–1.26)	.34	1.29 (0.95–1.75)	.10	1.64 (1.41–1.90)	<.01
120% LRL	1.34 (1.04–1.74)	.03	1.17 (1.02–1.34)	.03	1.28 (0.97–1.68)	.08	1.52 (1.33–1.74)	<.01

*: first trimester; **: third trimester; CI: confidence interval; GH: gestational hypertension; GDM: gestational diabetes mellitus; ICP: intrahepatic cholestasis of pregnancy; LRL: lower reference interval limit; OR: odds ratio; PE: preeclampsia; PPH: postpartum haemorrhage; TC: total cholesterol; TG: triglycerides; URL: upper reference interval limit.

## Discussion

In this population-based prospective study, the serum levels of TC, TG, HDL-C and LDL-C were all found significantly increased in the third trimester (Figure 1), which was consistent compared to other reports in the previous studies [3,16]. The increase of serum lipids is thought to be attributed to oestrogen stimulation and insulin resistance that are hallmarks of metabolic changes during pregnancy [17]. Besides, other maternal factors, such as pre-pregnancy BMI, and age, may also have impacts on lipid metabolism. Therefore, monitoring of serum lipid levels, beyond doubt, is particularly important during pregnancy [18] and it is crucial to establish trimester-specific serum lipids RIs for clinical practice. Meanwhile, the Hoffmann method successfully adopted in the present study required neither the recruitment of healthy subjects nor sophisticated computer data processing for determining RIs, showing potentially wide application in clinical laboratories.

With the cut-off values determined by the ROC analysis, all the serum lipids investigated showed much better potential in ruling out than ruling in pregnancy complications and perinatal outcomes (Table 2). However, the high NPVs were partially contributed by the low prevalence of these complications and adverse outcomes observed in the general pregnant population (Supplementary Table 2). To further investigate the associations between serum lipid levels and corresponding risks of the adverse pregnancy complications and perinatal outcomes, logistical regression analyses were performed (Tables 3 and 4). In particular, the first trimester TC, TG and LDL-C was found to be the most effective in predicting PE development. The risk for PE occurrence was nearly tripled if the TC level went up from 80% to 120% of its URL, suggesting that it may be beneficial to properly manage the increase of TC level when it reaches 80% URL to avoid PE development. A literature review by Ray et al. also showed that there was a consistent positive association between elevated maternal TG and the risk of PE in the previous case–control or prospective cohort studies [19]. Similarly, in a population-based cohort study, significantly increased pregnancy serum levels of TG and TC were found to be risk factors to PE [20]. Since the end of the first trimester, serum lipids start to increase in favour of maternal tissue lipid use as energy source, sparing glucose and amino acids for the foetus development. However, in the pregnant women with dyslipidaemia, increased serum lipids may induce extra oxidative stress *via* endothelial dysfunction and lead to increased PE pathogenesis [21]. Therefore, lipid measurements obtained in early pregnancy is helpful in identifying women at higher risk of developing preeclampsia.

A strong positive association was found between the first trimester TG and GDM, with the adjusted OR value of 1.85 (Table 3). It was believed that the pregnant women with GDM tend to have lower steroid hormones and sex hormone-binding globulin, which might contribute to development of hyperlipidaemia in diabetic pregnancy [21,22]. Similar to our result, Shen’s study [23] demonstrated that TG elevation throughout gestation conferred increased risk of GDM and hypertensive disorders of pregnancy. Coincidentally, a prediction model study of Sweeting [24] suggested that the first trimester maternal TG that was independent of BMI was the strongest lipid predictor for GDM.

It was previously reported that ICP is associated with dyslipidaemia, which may participate in the pathogenesis of the disease [25]. In our study, it was also found that the risk of ICP was more than doubled when the first trimester TG level was 20% higher than its URL (Table 3). In Zhang *et al.*’s study [26] with Chinese pregnant women, it was suggested that higher expressions of peroxisome proliferator-activated receptor γ and nuclear factor kappa B might disturb placental bile acid and serum lipids transportation, leading to fatal cholestasis which probably be one of the mechanism of ICP. In another study, the bile-acid-activated receptors TGR5 and farnesoid-X-receptor involved in lipid and glucose homoeostasis were also considered possible mechanisms for increased TG in the ICP patients [27].

A prospective study by Adank *et al.* [28] with 5702 pregnant women showed that early pregnancy TG level was associated with slight increase of risk of large-for-gestational age (LGA) (OR: 1.18, 95% CI: 1.07–1.29). Although maternal circulating TG does not directly cross the placenta, the presence of lipoprotein receptors, fatty acid–binding proteins, and different lipase activities in the placenta enable efficient transfer of maternal fatty acids to the foetus [29]. We observed similar finding that the OR value for macrosomia was 1.12 (95% CI: 0.66–1.92), although it was statistically insignificant (*p* = .67) which might be attributed to the relatively low macrosomia case number (*n* = 294).

Our logistic regression analysis showed that first- and third-trimester TC, TG and the third-trimester HDL-C were risk factors for PPH (Tables 3 and 4). More importantly, when third trimester TC concentration was higher than 120% URL, the risk of PPH occurrence was more than three-fold increased. Earlier studies suggested that dyslipidaemia was associated with endothelial dysfunction and disturbance of blood coagulation, the mechanisms of which could include production of proinflammatory interleukins and cytokines, fibrinogen, coagulation factors and impairment of fibrinolysis [30,31]. TG remnants have been shown to upregulate the endothelial expression of adhesion molecules, leading to endothelial monocyte adhesion and an enhanced inflammatory response [32]. These combined findings may help understanding the connections between dyslipidaemia and PPH mechanistically. Another population-based cohort study [33] reported that the risk of PPH attributable to atonic uterus was markedly increased in the obese pregnant group, highlighting the potential association between serum lipids and PPH. However, the direct relationship between serum lipids and PPH is still unclear, which requires further clinical and laboratory investigations.

Our study design has several strengths. This was a large population-based study with 16,489 pregnant women recruited. The serum samples were prospectively collected before occurrences of pregnancy complications or adverse perinatal outcomes. This experimental design allowed us to evaluate the associations between dyslipidaemia and the various complications and outcomes prospectively, providing direct evidence for their predictive values based on the population-based lipid screening profiles. In addition, the Hoffmann method was applied to estimate the RIs of maternal serum lipids with consistent results when compared with those derived from the conventional method, which further proved its merit and validity in pregnant population. Last but not least, the associations between the serum lipid levels during pregnancy and the various complications/adverse outcomes were revealed by multivariate logistic regression analysis in our study. This analytical strategy was proven to be an effective research tool and was successfully applied in another population-based study (*n* = 9911) which demonstrated that increased TG level posed higher risks of developing GDM or PE in pregnancy [34].

However, limitations still exit in the present study. Firstly, our cohort lacked population diversity. Given that China is a country characterised by diverse races and imbalanced regional economic development, a multicentre study involving different geographic areas can be more representative especially for the RI estimation. Similarly, the RIs obtained from current study may not be directly applied to non-Chinese populations due to differences in ethnicity, dietary habit, and even living environment [35,36]. Secondly, the pre-pregnancy lipid profiles were not assessed with our patients. Whether maternal weight control before pregnancy is associated with trimester lipid levels and pregnancy complications and/or outcomes remains unclear.

## Conclusion

In summary, incidence of gestational complications and adverse perinatal outcomes were positively associated with the maternal levels of TC, TG and LDL-C, and negatively associated with the level of HDL-C in our population-based prospective study. With the trimester-specific RIs established appropriately, the maternal serum lipids may be used as predictive and warning factors for some of the complications or adverse outcomes, even though they are more effective in ruling out than ruling in those diseases.

## Supplementary Material

Supplemental Material

## Data Availability

The authors confirm that the data supporting the findings of this study are available within the article and its supplementary materials.
