# Supplementary material for: Establishment of trimester-specific reference intervals of serum lipids and the associations with pregnancy complications and adverse perinatal outcomes: a population-based prospective study
Source: Ann Med. 2021 Sep 9;53(1):1632–41. doi: 10.1080/07853890.2021.1974082 (PMC8439224; doi:10.1080/07853890.2021.1974082)
Supplement: Supplemental Material [file IANN_A_1974082_SM9383.zip › Supplemental files/Lipid Supplementary Tables revise.docx]

**Supplemental Table 1** Diagnosis criteria of pregnancy complications and adverse prenatal outcomes

|  |  |
| --- | --- |
| Diseases | Definition |
| GH | Defined as blood pressure elevation [systolic blood pressure ≥ 140 mmHg or diastolic blood pressure ≥ 90 mmHg] at > 20 weeks’ gestation in the absence of proteinuria (1). |
| GDM | Before an oral glucose tolerance test (OGTT), every participant was requested for a 50 g glucose challenge test and serum glucose levels were assayed 1 h later. Subjects with positive results (glucose levels ≥7.8 mmol/L) were required to undergo a 75 g OGTT. Serum glucose levels during OGTT were measured at 0, 1 and 2 h, respectively. The normal values were fasting glucose <5.1 mmol/L, 1-h glucose <10.0 mmol/L and 2-h glucose <8.5 mmol/L. If one or more values equaled or exceeded the above thresholds, women were diagnosed as having GDM (2). |
| PE | New-onset hypertension (systolic blood pressure ≥140 mmHg or diastolic blood pressure ≥90 mmHg) and new-onset proteinuria (300 mg of protein in 24 h or a urine protein/creatinine ratio of 0.3 mg/dl) after 20 weeks of gestation, in a previously normotensive woman (1). |
| ICP | ICP is a pregnancy-specific disorder typically occurs in the third trimester characterized by pruritus and jaundice. Confirmation of diagnosis relied on abnormal liver function tests and raised maternal serum bile acids. Abnormal liver function tests included elevated levels of alanine aminotransferase, aspartate aminotransferase and/or gamma-glutamyl transpeptidase. The upper limits of total serum bile acids were 10–14 μmoles/L in postprandial state and 6–10 micromoles/L in fasting state. Exceeding the upper limits was an important diagnostic basis of ICP (3). |
| Macrosomia | Fetal birth weight ≥ 4000 g, regardless of gestational age (4). |
| PPH | Cumulative blood loss of ≥1,000 mL OR blood loss accompanied by signs and symptoms of hypovolemia within 24 hours following the birth process (5). |
| GH: gestational hypertension; GDM: gestational diabetes mellitus; PE: preeclampsia; ICP: intrahepatic cholestasis of pregnancy; PPH: postpartum hemorrhage. | |

**References**

1. Hypertension in pregnancy. Report of the American College of Obstetricians and Gynecologists' task force on hypertension in pregnancy. Obstet Gynecol. 2013;122(5):1122–31.

2. World Health Organization. Diagnostic criteria and classification of hyperglycemia first detected in pregnancy: a World Health Organization Guideline. Diabetes Res Clin Pract. 2014;103(3):341–63.

3. Williamson C, Geenes V. Intrahepatic cholestasis of pregnancy. Obstet Gynecol. 2014;124(1):120–33.

4. Macrosomia: ACOG Practice Bulletin, Number 216. Obstet Gynecol. 2020;135(1):e18-e35.

5. Practice Bulletin No. 183: Postpartum Hemorrhage. Committee on Practice Bulletins-Obstetrics. Obstet Gynecol. 2017;130(4):e168-e186

**Supplementary Table 2** Demographic data and basic statistics pf the serum lipids in patients with different outcomes

|  |  |  |  |
| --- | --- | --- | --- |
|  | Age | Pre-pregnancy BMI | TC |
| GH positive^*^ (N=317, 1.9%^***^) | 31 (29-35) | 23.23 (20.73-26.17) | 4.38 (3.94-4.84) |
| GH negative^*^ (N=16172) | 31 (29-34) | 21.23 (19.53-23.44) | 4.22 (3.79-4.70) |
| *p* value | 0.176 | <0.001 | <0.001 |
| GDM positive^*^ (N=1239, 7.5%^***^) | 33 (30-36) | 23.03 (20.86-25.64) | 4.37 (3.90-4.87） |
| GDM negative^*^ (N=15250) | 31 (29-34) | 21.12 (19.49-23.28) | 4.21 (3.79-4.69) |
| *p* value | <0.001 | <0.001 | <0.001 |
| PE positive^*^ (N=631, 3.8%^***^) | 32 (29-35) | 24.03 (21.23-26.95) | 4.45 (4.00-4.93) |
| PE negative^*^ (N=15858) | 31 (29-34) | 21.22 (19.53-23.34) | 4.21 (3.79-4.69) |
| *p* value | <0.001 | <0.001 | <0.001 |
| ICP positive^*^ (N=42, 0.3%^***^) | 31 (29-35) | 20.89 (19.45-23.73) | 4.08 (3.74-4.57) |
| ICP negative^*^ (N=16447) | 31 (29-34) | 21.26 (19.53-23.44) | 4.22 (3.79-4.70) |
| *p* value | 0.476 | 0.580 | 0.381 |
| Macrosomia positive^*^ (N=294, 1.8%^***^) | 32 (30-35) | 22.93 (21.09-25.43) | 4.21 (3.79-4.78) |
| Macrosomia negative^*^ (N=16195) | 31 (29-34) | 21.23 (19.53-23.44) | 4.22 (3.79-4.70) |
| *p* value | <0.001 | <0.001 | 0.965 |
| Macrosomia positive^**^ (N=294) | 32 (30-35) | 22.93 (21.09-25.43) | 6.20 (5.46-7.04) |
| Macrosomia negative^**^ (N=16195) | 31 (29-34) | 21.23 (19.53-23.44) | 6.38 (5.69-7.16) |
| *p* value | <0.001 | <0.001 | 0.004 |
| PPH positive^*^ (N=1251, 7.6%^***^) | 32 (29-35) | 22.04 (20.06-24.50) | 4.30 (3.83-4.79) |
| PPH negative^*^ (N=15238) | 31 (29-34) | 21.23 (19.53-23.38) | 4.21 (3.79-4.70) |
| *p* value | <0.001 | <0.001 | <0.001 |
| PPH positive^**^ (N=1251) | 32 (29-35) | 22.04 (20.06-24.50) | 6.28 (5.60-7.13) |
| PPH negative^**^ (N=15238) | 31 (29-34) | 21.23 (19.53-23.38) | 6.38 (5.69-7.16) |
| *p* value | <0.001 | <0.001 | 0.021 |
|  |  |  |  |

**Supplementary Table 2** (continued)

|  |  |  |  |
| --- | --- | --- | --- |
|  | TG | HDL-C | LDL-C |
| GH positive^*^ (N=317, 1.9%^***^) | 1.34 (0.88-1.62) | 0.02 (1.24-1.60) | 2.40 (1.99-2.76) |
| GH negative^*^ (N=16172) | 1.01 (0.79-1.32) | 1.48 (1.30-1.69) | 2.17 (1.82-2.56) |
| *p* value | <0.001 | <0.001 | <0.001 |
| GDM positive^*^ (N=1239, 7.5%^***^) | 1.27 (0.94-1.72) | 1.40 (1.23-1.63) | 2.35 (1.97-2.75) |
| GDM negative^*^ (N=15250) | 1.00 (0.78-1.30) | 1.48 (1.30-1.69) | 2.16 (1.82-2.55) |
| *p* value | <0.001 | <0.001 | <0.001 |
| PE positive^*^ (N=631, 3.8%^***^) | 1.23 (0.93-1.61) | 1.39 (1.22-1.61) | 2.44 (2.03-2.86) |
| PE negative^*^ (N=15858) | 1.00 (0.79-1.32) | 1.48 (1.30-1.69) | 2.17 (1.82-2.56) |
| *p* value | <0.001 | <0.001 | <0.001 |
| ICP positive^*^ (N=42, 0.3%^***^) | 1.01 (0.82-1.30) | 1.39 (1.28-1.53) | 2.11 (1.87-2.53) |
| ICP negative^*^ (N=16447) | 1.01 (0.79-1.33) | 1.48 (1.30-1.69) | 2.17 (1.82-2.57) |
| *p* value | 0.576 | 0.034 | 0.600 |
| Macrosomia positive^*^ (N=294, 1.8%^***^) | 1.11 (0.84-1.46) | 1.42 (1.22-1.60) | 2.27 (1.87-2.60) |
| Macrosomia negative^*^ (N=16195) | 1.01 (0.79-1.32) | 1.48 (1.30-1.69) | 2.17 (1.82-2.57) |
| *p* value | <0.001 | <0.001 | 0.260 |
| Macrosomia positive^**^ (N=294) | 3.10 (2.58-3.79) | 1.68 (1.48-1.90) | 3.25 (2.60-3.95) |
| Macrosomia negative^**^ (N=16195) | 2.87 (2.33-3.58) | 1.78 (1.55-2.03) | 3.43 (2.80-4.10) |
| Prevalence, % |  | | |
| *p* value | <0.001 | <0.001 | 0.003 |
| PPH positive^*^ (N=1251, 7.6%^***^) | 1.10 (0.84-1.48) | 1.43 (1.26-1.65) | 2.26 (1.88-2.67) |
| PPH negative^*^ (N=15238) | 1.00 (0.79-1.32) | 1.48 (1.30-1.69) | 2.17 (1.82-2.56) |
| *p* value | <0.001 | <0.001 | <0.001 |
| PPH positive^**^ (N=1251) | 3.12 (2.48-3.84) | 1.69 (1.46-1.92) | 3.37 (2.70-4.06) |
| PPH negative^**^ (N=15238) | 2.86 (2.33-3.57) | 1.79 (1.56-2.03) | 3.43 (2.80-4.11) |
| *p* value | <0.001 | <0.001 | 0.029 |

All lipid values were presented as median (25^th^-75^th^ percentile). *: first trimester; **: third trimester; ***: prevalence of each complication (%) in the recruited population; GH: gestational hypertension; GDM: gestational diabetes mellitus; PE: preeclampsia; ICP: intrahepatic cholestasis of pregnancy; PPH: postpartum hemorrhage; BMI: body mass index; TC: total cholesterol; TG: triglycerides; HDL-C: high-density lipid cholesterol; LDL-C: low-density lipid cholesterol.

**Supplementary Table 3** Logistic regression analysis of the risk of age and pre-pregnancy BMI for pregnancy complications and adverse pregnancy outcomes

|  |  |  |  |  |  |  |  |  | |  | | |  |  | |  |  |
| --- | --- | --- | --- | --- | --- | --- | --- | --- | --- | --- | --- | --- | --- | --- | --- | --- | --- |
|  | Variable | GH^*^ | |  | GDM^*^ | |  | PE^*^ | | | | |  | ICP^*^ | | |  |
|  |  | OR (95% CI) | *p* value |  | OR (95% CI) | *p* value |  | OR (95% CI) | *p* value | |  | OR (95% CI) | | | *p* value | | |
| TC | Age | 1.00 (0.97-1.03) | 0.98 |  | 1.11 (1.09-1.12) | <0.01 |  | 1.03 (1.01-1.05) | 0.01 | |  | 1.04 (0.96-1.12) | | | 0.34 | | |
|  | BMI | 1.14 (1.11-1.17) | <0.01 |  | 1.14 (1.13-1.16) | <0.01 |  | 1.20 (1.18-1.23) | <0.01 | |  | 0.97 (0.88-1.07) | | | 0.57 | | |
| TG | Age | 1.00 (0.97-1.03) | 0.81 |  | 1.10 (1.09-1.12) | <0.01 |  | 1.02 (1.00-1.04) | 0.02 | |  | 1.03 (0.95-1.11) | | | 0.47 | | |
|  | BMI | 1.14 (1.11-1.17) | <0.01 |  | 1.14 (1.12-1.15) | <0.01 |  | 1.20 (1.18-1.22) | <0.01 | |  | 0.95 (0.86-1.05) | | | 0.32 | | |
| LDL-C | Age | 1.00 (0.97-1.03) | 1.00 |  | 1.11 (1.09-1.12) | <0.01 |  | 1.03 (1.01-1.05) | 0.01 | |  | 1.04 (0.96-1.12) | | | 0.32 | | |
|  | BMI | 1.14 (1.11-1.17) | <0.01 |  | 1.14 (1.13-1.16) | <0.01 |  | 1.20 (1.18-1.23) | <0.01 | |  | 0.98 (0.88-1.08) | | | 0.63 | | |
| HDL-C | Age | 1.00 (0.97-1.03) | 0.96 |  | 1.11 (1.09-1.12) | <0.01 |  | 1.03 (1.01-1.05) | 0.01 | |  | 1.04 (0.96-1.12) | | | 0.33 | | |
|  | BMI | 1.14 (1.11-1.17) | <0.01 |  | 1.14 (1.12-1.16) | <0.02 |  | 1.20 (1.18-1.23) | <0.01 | |  | 0.97 (0.88-1.08) | | | 0.59 | | |

**Supplementary Table 3** (continued)

|  |  |  |  |  |  |  |  |  |  |  |  |  |  |
| --- | --- | --- | --- | --- | --- | --- | --- | --- | --- | --- | --- | --- | --- |
|  | Variable |  | Macrosomia^*^ | |  | PPH^*^ | |  | Macrosomia^**^ | |  | PPH^**^ | |
|  |  |  | OR (95% CI) | *p* value |  | OR (95% CI) | *p* value |  | OR (95% CI) | *p* value |  | OR (95% CI) | *p* value |
| TC | Age |  | 1.04 (1.01-1.07) | 0.01 |  | 1.05 (1.03-1.06) | <0.01 |  | 1.04 (1.01-1.07) | 0.01 |  | 1.05 (1.03-1.06) | <0.01 |
|  | BMI |  | 1.14 (1.11-1.17) | <0.01 |  | 1.07 (1.06-1.09) | <0.01 |  | 1.14 (1.10-1.17) | <0.01 |  | 1.08 (1.06-1.09) | <0.01 |
| TG | Age |  | 1.04 (1.01-1.07) | 0.01 |  | 1.04 (1.03-1.06) | <0.01 |  | 1.04 (1.01-1.07) | 0.01 |  | 1.04 (1.03-1.06) | <0.01 |
|  | BMI |  | 1.13 (1.10-1.17) | <0.01 |  | 1.07 (1.05-1.09) | <0.01 |  | 1.14 (1.10-1.17) | <0.01 |  | 1.07 (1.06-1.09) | <0.01 |
| LDL-C | Age |  | 1.04 (1.01-1.07) | 0.01 |  | 1.05 (1.03-1.06) | <0.01 |  | 1.04 (1.01-1.07) | 0.01 |  | 1.05 (1.03-1.06) | <0.01 |
|  | BMI |  | 1.14 (1.11-1.17) | <0.01 |  | 1.07 (1.06-1.09) | <0.01 |  | 1.14 (1.10-1.17) | <0.01 |  | 1.07 (1.06-1.09) | <0.01 |
| HDL-C | Age |  | 1.04 (1.01-1.07) | 0.01 |  | 1.05 (1.03-1.06) | <0.01 |  | 1.04 (1.01-1.07) | 0.01 |  | 1.04 (1.03-1.06) | <0.01 |
|  | BMI |  | 1.13 (1.10-1.17) | <0.01 |  | 1.07 (1.06-1.09) | <0.01 |  | 1.13 (1.10-1.17) | <0.01 |  | 1.07 (1.05-1.09) | <0.01 |

*: first trimester; **: third trimester; BMI: body mass index; GH: gestational hypertension; GDM: gestational diabetes mellitus; PE: preeclampsia; ICP: intrahepatic cholestasis of pregnancy; PPH: postpartum hemorrhage; HDL-C: high-density lipid cholesterol; LDL-C: low-density lipid cholesterol; CI: confidence interval; OR: odds ratio.
