# Supplementary figures and images for: Establishment of trimester-specific reference intervals of serum lipids and the associations with pregnancy complications and adverse perinatal outcomes: a population-based prospective study
Source: Ann Med. 2021 Sep 9;53(1):1632–41. doi: 10.1080/07853890.2021.1974082 (PMC8439224; doi:10.1080/07853890.2021.1974082)

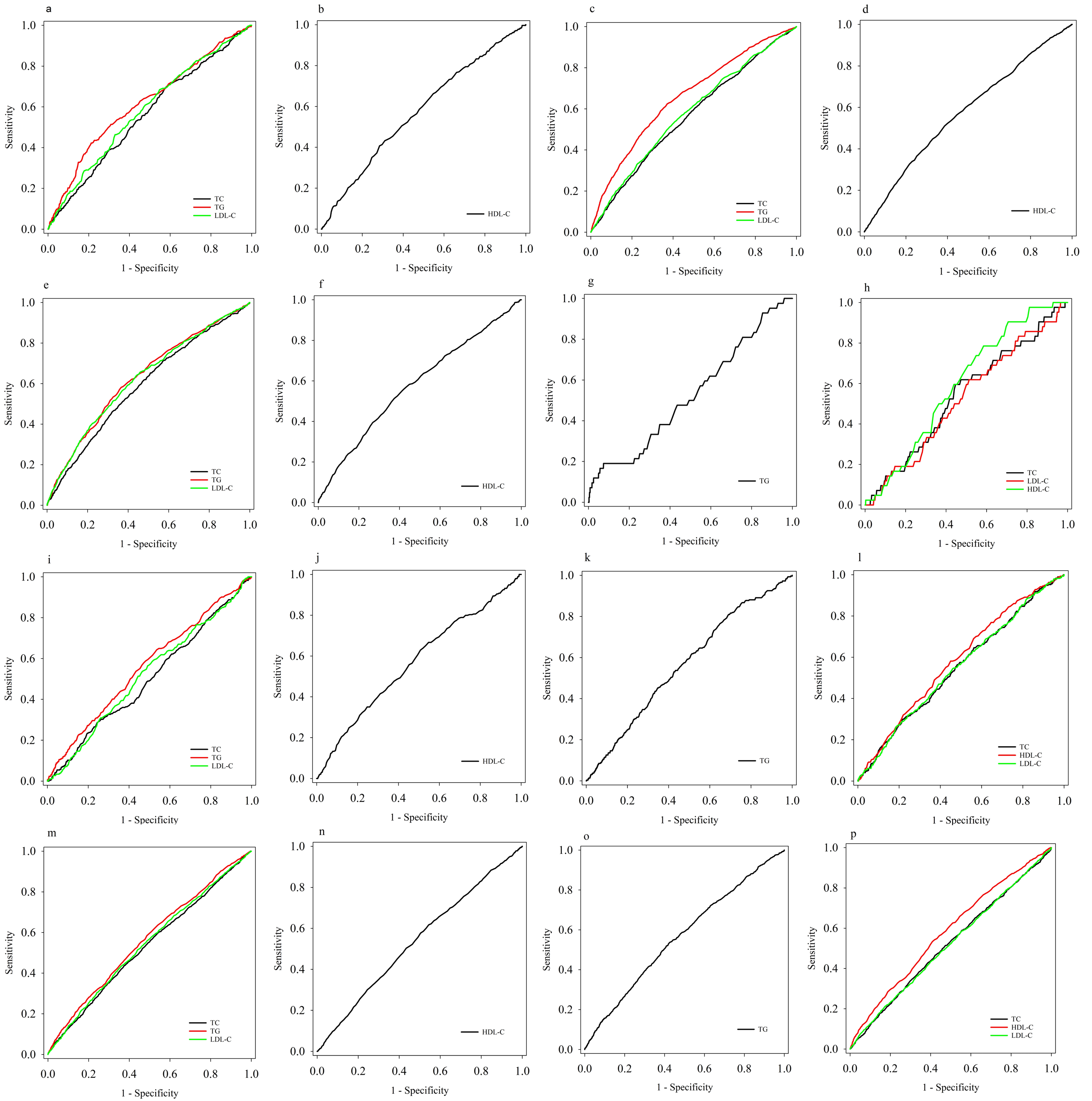

Supplement: Supplemental Material [file IANN_A_1974082_SM9383.zip › Supplemental files/Supplementary Figure1.tif]
